# Supplementary material for: Are People with Obesity Attracted to Multidisciplinary Telemedicine Approach for Weight Management?
Source: Nutrients. 2022 Apr 11;14(8):1579. doi: 10.3390/nu14081579 (PMC9027321; doi:10.3390/nu14081579)
Supplement: Supplementary file 1 [file nutrients-14-01579-s001.zip › nutrients-1655391-supplementary.pdf]

Participating in this survey, I give my consent to the use of the data I entered anonymously: YES / NO

Please indicate your gender *

F

M

Insert your age *___

Enter your educational level *

None

Primary school

Secondary school

University

Doctorate, Master, Specialty

Work activity *

Student

Employee (public offices, banks, shops, services, etc.)

Worker / Technician

Health worker (healthcare assistant, nurse, doctor)

Entrepreneur

Freelance

Retired

Housewife

Unemployed

Enter your postcode:________

Enter your weight (kg)*:__________

Enter your height (m)*:______

Do you suffer from diseases related to excess weight (eg diabetes, respiratory diseases, hypertension, orthopedic disabilities, etc.)?

Yes

No

You own an electronic tool? (*multiple choices*)

Yes, smart-phone

Yes, Tablet

Si, PC

No

Have you cancelled or postponed any scheduled clinical assessments during pandemic?

Yes

No

In the impossibility of a visit during this period, have you contacted your doctor for the management of your complications in any other way?

Yes, by telephone

Yes, by email

Yes, by WhatsApp or phone message

Yes, by video consulting

No, never

Do you think that in this period a remote medical video consultation could make you run less health risks?

Yes

No

I don’t know

Do you think your doctor can understand your health through a video consultation?

Yes

No

I don’t know

Yes, but only if he has already met me during a face-to-face visit

Would you be like to start an online multidisciplinary intervention for weight management?

Yes

Yes, with known health professional

No

I don’t know

Do you think that the cost of the online lifestyle intervention should be provided by the national health system?

Yes

No

Would you be willing to do it for a fee?

Yes

No

It depends on the cost

Would you be like to continue an online multidisciplinary intervention for weight management, stpped because of pandemic?

Yes

Yes, but only with known health professional

No

I don’t know

Do you think that the cost of this intervention should be provided by the national health system?

Yes

No

Would you be willing to do it for a fee?

Yes

No

It depends on the cost

Did you have to take psychological support during the pandemic?

Yes

No

Did you have to stop a psychological support during the pandemic?

Yes

No

Do you think a psychological video consultating could be useful to better manage emotions / stress and nervous hunger in your daily life?

Yes

No

Yes, but only with known health professional

I don't know

Would you feel comfortable talking about your emotions to the psychologist via a screen?

Yes

No

Yes, but only with known health professional

I don't know

In your opinion, what frequency should psychological support have?

Once a week

Twice a week

Whenever I feel the need

Would you undergo a remotely delivered psychological intervention?

Yes

No

Yes, but only with known health professional

I don't know

Have you already done diets to manage your weight?

Yes, at least one

Yes, more than 3

Yes, more than 5

No, never

Did you have to stop scheduled visits for diet and nutrition during this time?

Yes

No

Would you will join an online nutritional intervention?

Yes

No

Yes , but only with known health professional

I don’t know

Which of the following modalities would you like to better follow a diet?

Individual video consulting once a week

Group video consulting one a week

Individual video consulting, but only when I feel the need

Video recordings, educational / informative material on diet-nutrition / health, to view if necessary

Do you regularly practice physical activity? If "YES" what type (for example: fitness in the gym, swimming-water aerobics, pilates, postural gymnastics, walking outdoors)?

Yes (indicate the main physical activity): …………………………………………………..

No

Have you ever attended “remote” physical activity classes (for example: postural gymnastics, pilates, yoga, etc)?

Yes, with real-time online lessons

Yes, with recorded lessons

No, never

Would you like to practise physical activity, online, supervised by an exercise physiologist??

Yes

No

I don’t know

What could prevent you from exercising online? (as many answers as possible)

- laziness

- the lack of proper space at home

- the lack of a compter-tablet-smartphone to connect

- difficulty to use the electronic tools to connect

-fear of getting

- nothing, I would gladly do it

Which of the following modalities would you prefer to practise physical activity at home?

Real-time online group lessons, once a week

Real-time online individual lessons, once a week

Reception of training tables and one-to-one meetings via the video platform with an exercise physiologist, once a week
